# Griscelli Syndrome Type 2: Comprehensive Analysis of 149 New and Previously Described Patients with RAB27A Deficiency

**DOI:** 10.1007/s10875-024-01842-2

**Published:** 2024-11-28

**Authors:** Jesmeen Maimaris, Adriel Roa-Bautista, Mahreen Sohail, Claire Booth, Chiara Cugno, Lenka Chenchara, Tawfeg Ben Omran, Yael Hacohen, Ming Lim, Kimberly Gilmour, Gillian Griffiths, Kanchan Rao, Reem Elfeky, Maaike Kusters

**Affiliations:** 1 University College London UCL Institute of Immunity and Transplantation, London, UK; 2 Paediatric Immunology Department, Great Ormond Street Hospital for Children National Health Service (NHS) Foundation Trust, London, UK; 3 Immunology department, Manchester University Hospital National Health Service (NHS) Foundation Trust, Manchester, UK; 4 Paediatric department, Barts Hospital National Health Service (NHS) Foundation Trust, London, UK; 5 UCL Great Ormond Street Institute of Child Health, London, UK; 6 Advanced Cell Therapy Core, Research Department, Sidra Medicine, Doha, Qatar; 7 Pediatric Oncology, Haematology and Bone Marrow Transplantation Unit, Sidra Medicine, Doha, Qatar; 8 Genetics and Genomic Medicine Department, Sidra Medicine, Doha, Qatar; 9 Department of Paediatric Neurology, Great Ormond Street Hospital for Children National Health Service (NHS) Foundation Trust, London, UK; 10 Children’s Neurosciences, Evelina London Children’s Hospital Neurosciences Department, London, UK; 11 Faculty of Life Sciences and Medicine, King’s College London, London, UK; 12 Cambridge Institute for Medical Research, University of Cambridge, Cambridge, UK; 13 Department of Bone Marrow Transplantation, Great Ormond Street Hospital for Children NHS Trust, London, UK

## Abstract

Griscelli syndrome type 2 (GS2) is a rare, life-threatening immunodysregulatory disorder characterised by impaired cytotoxic activity leading to susceptibility to haemophagocytic lymphohistiocytosis (HLH) and hypopigmentation. We completed a literature review and analysis of clinical data of 149 patients with GS2 including 8 new patients.

We identified three founder mutations which show diverse phenotypic profiles (*RAB27A* c.244 C > T, p.R82C, c.514_518delCAAGC, p.Q172NfsX2, c.550 C > T, p.R184X). The most common presentation was HLH (119/149, 80%), with high proportion of central nervous system involvement (68/149, 46%). Features of partial albinism were present in 105 of 149 cases (70%). Hypopigmentation can be absent in GS2 and should not exclude the diagnosis. Patients with biallelic protein truncating variants (PTV) were more likely to have systemic HLH (44/56, 79%) and partial albinism (45/56, 80%), in comparison to hypomorphic variants (9/41, 22%; 20/41, 49%). Patients with hypomorphic variants presented later (5.4 years cf. 0.4 years, *p* = < 0.0001) and were more likely to have isolated CNS HLH (2% cf. 42%, *p* = 0.001).

Mortality was high in the cohort (50/149, 34%). Survival of cases post-HLH who underwent transplantation is superior to un-transplanted patients, suggesting adequate HLH control followed by early HSCT is highly beneficial. Mortality was reduced in HSCT recipients versus the un-transplanted group where follow-up data was available (14% compared to 58%).

Asymptomatic cases identified through family history/genetic screening may benefit from pre-emptive HSCT, but access and development of robust functional testing are required. High mortality related to HLH remains concerning and emphasises the need for improved molecular characterisation and clinical prognostic factors to guide management decisions.

**Supplementary Information:**

The online version contains supplementary material available at 10.1007/s10875-024-01842-2.

## Introduction

Griscelli syndrome is a rare autosomal recessive disorder characterized by partial albinism first described by the French paediatrician Claude Griscelli in 1978 [1]. Since then, three distinct types of the syndrome have been genetically defined, with biallelic *RAB27A* genetic mutations associated with Griscelli syndrome type 2 (GS2), (OMIM: 607624). This is a clinically heterogeneous syndrome primarily featuring hypopigmented skin, silvery-grey hair and cellular immunodeficiency with propensity to haemophagocytic lymphohistiocytosis (HLH) [2, 3]. It is distinct from the other hypopigmentary Griscelli syndromes in its association with HLH: type 1 (OMIM: 214450) caused by biallelic *MYO5A* mutations, and type 3 (OMIM: 609227) caused by biallelic *MLPH* pathogenic mutations.

The *RAB27A* gene is located on chromosome 15q and contains 7 exons, with the first 2 being non-coding. *RAB27A* encodes a small 95 kDa GTPase widely expressed in secretory cells and tissues, including melanocytes and granulocytes. Rab27a acts as a molecular switch by cycling between an active guanosine triphosphate (GTP) binding state, and the inactive hydrolysing GTP to guanosine diphosphate (GDP), translating it to its ‘inactive’ form. Two switch regions have been shown to change conformation upon GDP or GTP binding; switch I localised to 10 amino acids in exon 3, and switch II region, approximately 17 amino acids straddling exon 4 and 5 [4]. Biallelic loss of function mutations are thought to abolish *RAB27A* expression resulting in reduced vesicular formation and transport [5]. Rab27a protein is critical for multi-vesicular endosome trafficking, fusion and docking at the plasma membrane [6–8]. Specifically, it is required for the trafficking of melanosomes in melanocytes and facilitating the exocytosis of granules from cytotoxic T cells (CTL), neutrophils and natural killer (NK) cells [9, 10] which underlies the clinical manifestations of hypopigmentation and immune deficiency. Previous studies have sought to characterise critical protein binding sites of Munc13-4 and Slp2a, its effector protein, on Rab27a, particularly in the absence of specific functional domains of the protein. These have identified hypomorphic mutations of *RAB27A* which demonstrate potential melanophilin (Mlph) binding sites including R184 which disrupt granule formation in the absence of partial albinism [6, 11–13]. In *RAB27A* deficiency, intracellular cytotoxic granule release is disrupted resulting in failure to clear infected cells, with excessive activation of macrophages and failure to terminate the immune response [6, 12]. To confirm disruption of the cytotoxic granule pathway, a granule release assay can be used. This is a flow cytometric test which detects a cell surface protein CD107a, as a marker of functional cytotoxic granule formation in lymphocytes [14, 15]. The assay is specific in identifying lymphocyte defects critical for degranulation and exocytosis in lymphocytes such as Rab27a, Syntaxin 11, Munc 13 − 4, and Munc 18 − 2; the absence of these proteins are known to cause HLH [14, 15]. This functional assay can support the diagnosis in affected patients particularly where a genetic diagnosis is not yet made.

Mortality of patients affected by GS2 is high due to the predisposition to HLH [16]. The precise triggers for the development of HLH are varied but may not be identified. HLH is a life-threatening immunological disorder characterised by hyperinflammation and release of inflammatory cytokines, with fever, hepatosplenomegaly and cytopenia [17, 18]. Treatment protocols featuring immunosuppressive and cytotoxic medication have been developed to optimise outcomes in patients [19–21]. Haematopoietic stem cell transplantation (HSCT) is the only curative option for patients with GS2 [22–24].

Despite the many case reports of the clinical manifestations in GS2, several unresolved questions remain. Herein, we analyse the genetic, phenotypic features and outcomes of reported patients with GS2 in the literature, including 8 unpublished cases identified in our patient cohort [2, 3, 6, 11–13, 16, 22–68].

## Methods

We aimed to collate all patients with GS2 at our centre and in the literature to determine collective clinical features and patient outcomes. A systematic search was carried out utilizing the PubMed/MEDLINE database up to November 2023. The key search terms employed were “Griscelli syndrome type 2, Griscelli, RAB27A, haemophagocytic lymphohistiocytosis, hemophagocytic lymphohistiocytosis, HLH” aimed at identifying pertinent articles published in the English language and indexed in this comprehensive database. A screening process was conducted through two independent co-authors (ARB, MS). Studies using animal models, in-vitro models, and articles in languages other than English were excluded. Articles that were duplicates or lacked clinical features relevant to GS2 were excluded. Data collection included the following parameters: epidemiological features, clinical symptoms, laboratory and molecular data of the patients, including treatment information where available. Patient ethnicity or race was reported as originally described in papers. We also included 8 patients with GS2 from our centre who were not previously reported (P1-8).

### Statistical Analysis

Descriptive statistics were reported for quantitative data and performed using Graphpad Prism v10 (La Jolla, CA, USA). For variables with normal distribution, median and interquartile ranges (IQR) were reported. Associations between variables and clinical characteristics were compared with the use of the chi-square test (categorical variables) or unpaired t-test (continuous variables).

### Haplotype Analysis

Multipoint linkage and LOD scores were performed using the Superlink-Online SNP 1.1 [69]. For the parametric linkage analysis, fully penetrant autosomal recessive inheritance was assumed with a disease allele frequency of < 0.001. Suggestive linkage peaks with LOD scores > 2 analysed with SNP markers were selected from the whole exome sequencing variants of probands, where whole genome sequencing data were available.

### Granule Release Assay

Where available, patient-derived cells were interrogated for their ability to release cytotoxic granules by detecting the presence of CD107a, a cell surface protein on CTL and/or NK cells. Whole-blood samples (5–10 mL) were collected in EDTA-containing vials. A healthy control sample was processed in parallel to each patient sample. Peripheral blood mononuclear cells were separated from whole blood and stimulated overnight with IL-2. The cells were stimulated with PHA or anti-CD3 antibody in the presence of anti-CD107a antibody conjugated with FITC for 2 h, then stained with cell surface markers and analysed by flow cytometry. An abnormal result is reported when the increase in percentage of CD107a between stimulated and unstimulated samples was < 0.5% for cytotoxic T cells after anti-CD3 stimulation and/or < 5% for NK cells after PHA stimulation [70].

## Results

### Literature Review

In total, 114 articles were identified from the initial search, with 2 added after review. Following exclusion for duplicates or insufficient clinical details, 54 articles met the criteria and were deemed suitable for inclusion. The literature search identified 141 individual case reports of GS2 published in the literature until November 2023. In addition, 8 patients not previously described in the literature were identified from Great Ormond Street Hospital, London, United Kingdom for inclusion in the study following genetic diagnosis of *RAB27A* deficiency.

### Demographic Characteristics

In total, 149 patients were included in the study. The median age at GS2 diagnosis was 1.5 years, with a wide range spanning from 10 days to 42 years. 74 were males (50%), 57 were females (38%), and data on sex were unavailable for 18 cases (12%). Patients of Qatari descent were most frequently found in our study cohort (n = 20, 14%), followed by Turkish descent (n = 15, 10%). Asymptomatic molecular diagnosis was made based on positive family history in 12 cases (8%). The demographic data of all patients is summarised in Table 1.

**Table 1:** Demographic data of patients with RAB27A deficiency. Number of samples is denominator where data were available. Median with interquartile range (IQR) shown in age of onset/death)

Parameters (number of patients)	Results (%, or IQR)
Study total:	149 patients
Sex ratio, Male: Female	74:57 (50:38)
Age of onset (years)	1.5 (0.3-5)
Consanguinity	97 (65.1%)
Age of HLH diagnosis (years)	1.4 (0.4–4.1)
Age of death (years)	4.75 (0.96-10)
Overall mortality	50 (33.5%)
Late onset (≥ 10y)	16 (10.7%)
Molecular diagnosis:	139 patients
Protein-truncating variants (PTV)	56 (37.6%)
Hypomorphic variants (Missense)	41 (27.5%)
Other (compound PTV + missense)	42 (28.1%)

### Molecular Analysis

**Fig. 1:**
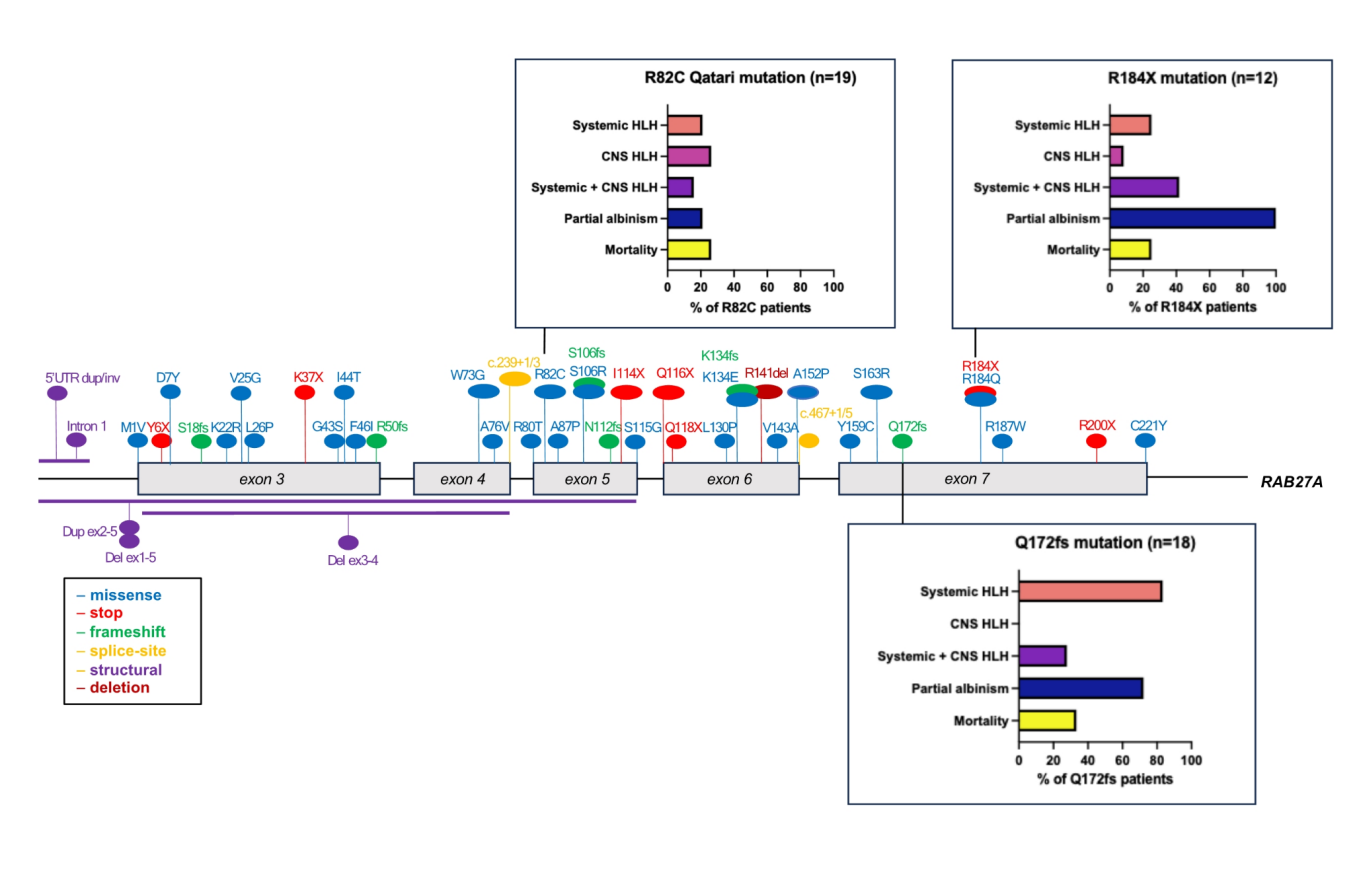
*RAB27A* biallelic pathogenic variants. Founder mutations with phenotypic features depicted in bar charts. The first 2 exons of *RAB27A* are non-coding

Genomic reports were available in 139 patients (93%). Overall, 56 distinct pathogenic sequence variants of *RAB27A* were identified, including intronic and structural variants. The mutations were distributed throughout the *RAB27A* gene, with several mutational hotspots identified (Fig. 1 and Supplemental Table 1). Analysis of haplotype blocks and literature review identified 3 potential founder mutations where genetic data were available: *RAB27A* c.244 C > T p.Arg82Cys, c.514_518del p.Gln172fs, c.550 C > T, p.Arg184X (NM_004580) [41, 71]. The *RAB27A* c.244 C > T p.Arg82Cys homozygous variant was only seen in Qatari patients. A shared haplotype block comprising 82 kilobases across the c.244 C > T p.Arg82Cys variant in 4 unrelated (Qatari) pedigrees. Shared haplotype blocks of 126 kilobases were found in 3 unrelated pedigrees across the c.514_518del p.Gln172fs variant and 5 unrelated patients with the c.550 C > T, p.Arg184X variant, suggestive of single recombination events from different common ancestors.

Figure 1 shows the three founder mutations identified with different phenotypic profiles and outcomes. Parental consanguinity was reported in nearly two-thirds of the cases reviewed (n = 97, 65%) with biallelic mutations mostly homozygous, indicative of consanguinity and founder effects.

### Clinical Manifestations

**Fig. 2:**
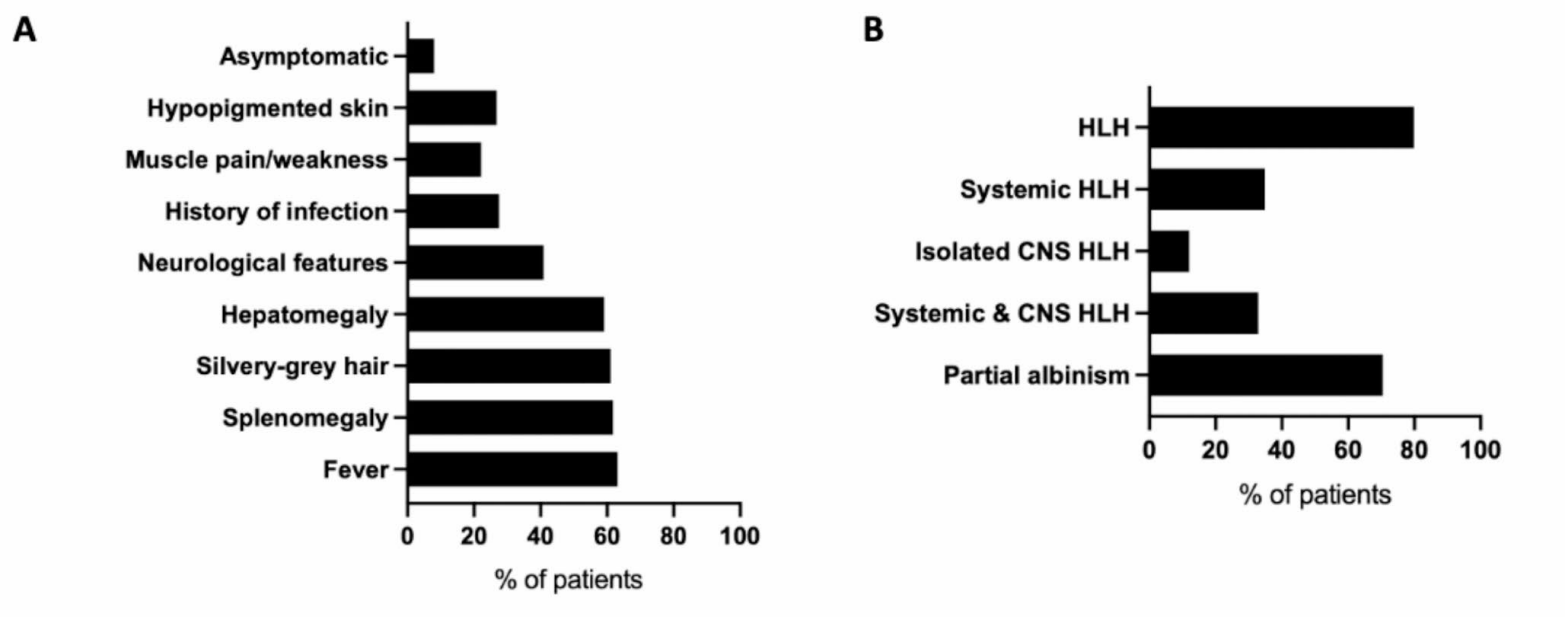
**A**: Frequency of occurrence of clinical features. Values represent percentages of total cases (n = 149). **B**: Frequency of occurrence of diagnoses. Values represent percentages of total cases (n = 149)

Clinical features were described in all 149 patients reported. Presenting clinical features included fever (n = 94, 63%), splenomegaly (n = 92, 62%), hepatomegaly (n = 88, 59%), neurological features (n = 61, 41%) and muscle weakness or myalgia (n = 33, 22%), (Fig. 2A). Neurological features include signs such as ataxia, strabismus (n = 55, 37%), seizures (n = 21, 14%), and developmental delay or cognitive dysfunction (n = 15, 10%), A large proportion of patients had silvery-grey hair (n = 75, 50%) with a smaller number having hypopigmented skin (n = 32, 21%) (Fig. 2A). A minority of patients were asymptomatic (n = 12, 8%), having received molecular diagnosis following positive family history. A history of infection was noted in a quarter of the cases evaluated (n = 40, 27%), with more than half reporting frequent viral infections (Fig. 2A).

Most patients developed HLH (n = 119, 80%). Information about HLH status was unavailable for 12 cases (8%) (Fig. 2B). Among the patients with HLH, nearly half presented with systemic HLH only (n = 52, 35%), combined systemic and central nervous system (CNS) HLH (n = 49, 33%), with the remaining cases presenting with isolated CNS HLH (n> = 18, 12%) HLH (Fig. 2B). HLH triggers were not adequately described in the literature to allow comment on this. Partial albinism was described in most patients as defined by the presence of one of the following: features of hypopigmented skin, silvery-grey hair, and uneven melanin pigmentation of the hair shaft detected by light microscopy (n = 105, 70%). 31 patients (21%) did not have any features of partial albinism. 5 patients in the cohort developed lymphoma which was successfully treated with chemotherapy (3%), with 2 specifically EBV-associated large B cell lymphoma [57, 58, 68].

### Genotype-Phenotype Correlation

**Fig. 3:**
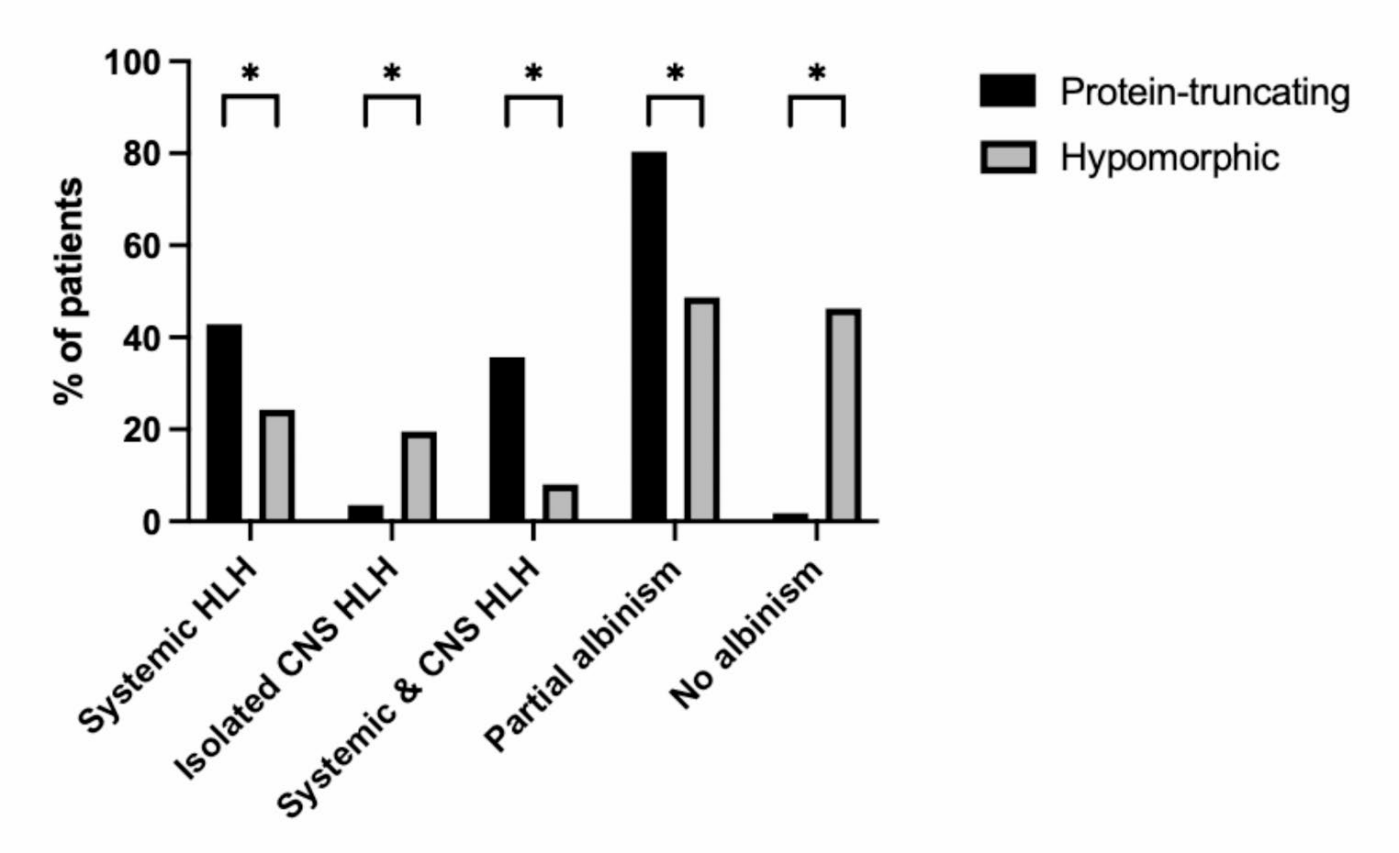
Frequency of clinical features according to genotype groups. Compound variant types were excluded * denotes *p*-value < 0.05 from unpaired t-tests

**Fig. 4:**
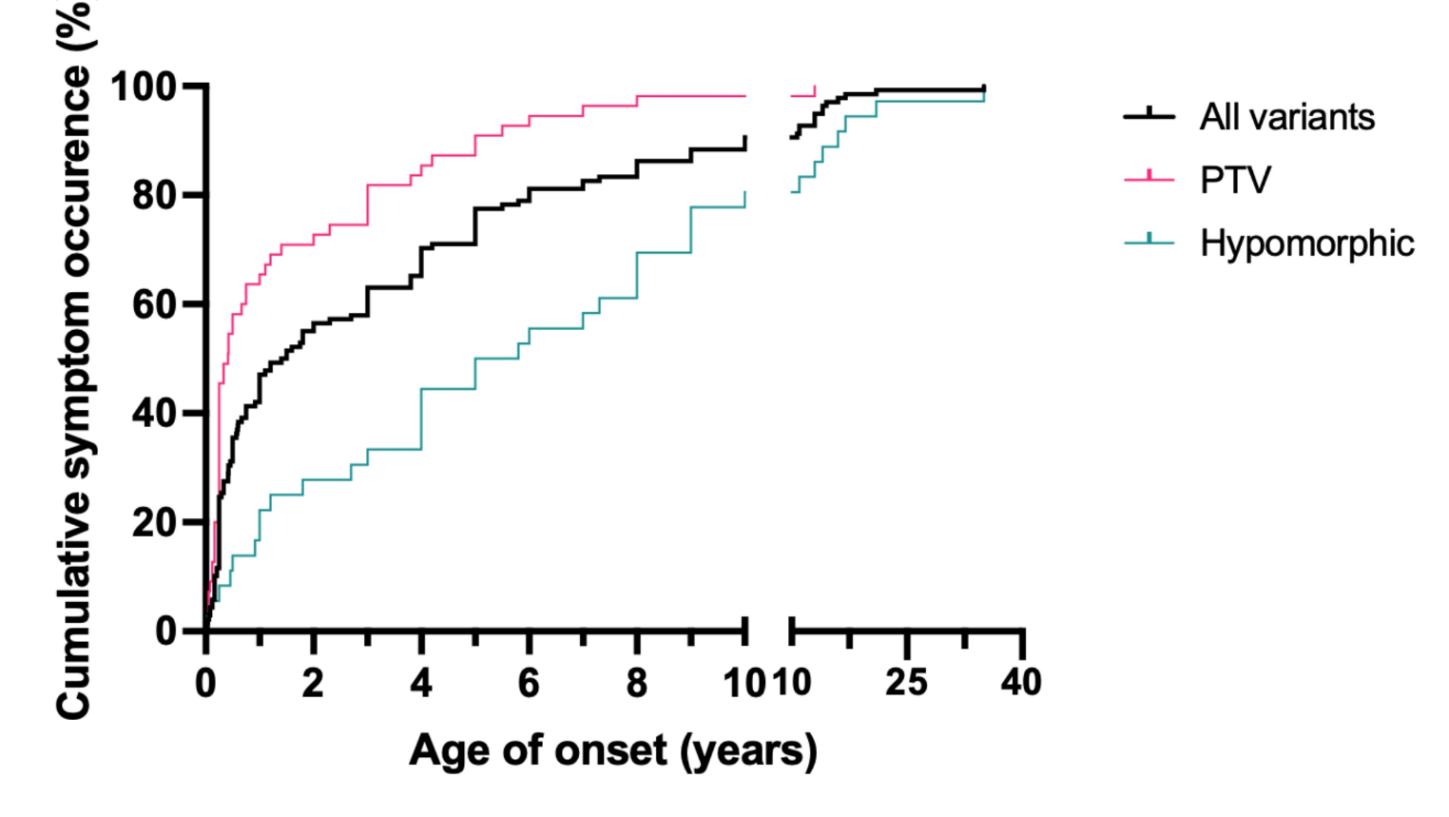
Onset of any symptoms in patients with PTVs and hypomorphic variants in Griscelli syndrome type 2

To analyse genotype-phenotype correlation, the patients were divided into those with: (1) protein-truncating variants (PTV) (n = 56; 38%), expected to abolish Rab27a protein expression and function, and (2) hypomorphic variants (n = 41; 28%) such as compound heterozygous or biallelic missense mutations that are predicted to have residual Rab27a expression. Our analysis found patients with biallelic PTV were more likely to have HLH, particularly systemic HLH (24/56, 43%) and partial albinism (45/56, 80%), in comparison to hypomorphic variants (9/41, 22%; 20/41, 49%) (Fig. 3). However, isolated CNS HLH occurred more frequently in patients with hypomorphic variants (8/41, 20% compared to 2/56, 4%, *p* = 0.002). Only 1 patient with biallelic PTV did not have any features of partial albinism, compared to 19 patients with hypomorphic variants (2% compared to 46%, *p* = 0.001). The median age of onset of symptoms was 1.5 years (IQR = 0.3-5 years), and patients with PTVs presented at an earlier age (median age: 0.4 years, IQR = 0.25–2.7 years) than patients with biallelic hypomorphic mutations (median age: 5.4 years, IQR = 1.65-9 years) (X^2^ = 24.54, *p* = < 0.0001) (Fig. 4).

The most common pathogenic variant was the Qatari homozygous variant c.244 C > T, p.R82C which was identified in 19 of the study cohort (13%). This variant did not have typical features of GS2 as only 4 patients presented with systemic HLH (21%) and 4 patients had features of partial albinism (21%), while 5 had CNS HLH (26%). This contrasts with the founder variant c.514–518delCAAGC, p.Q172NfsX2 (n = 18) which had 15 patients presenting with systemic HLH (83%) and 13 patients with partial albinism (72%). Isolated CNS HLH was not found in any patients with the Q172fs founder variant and co-occurred with systemic HLH in 5 cases (28%) (Fig. 1). The Qatari variant R82C also differed from the c.550 C > T, p.R184X homozygous variant which is found in 12 patients in the study cohort. All 12 patients presented features of partial albinism, 3 developed systemic HLH (25%) and 5 CNS HLH (41%) (Fig. 1).

### Characteristics of Late-Onset Presentation

**Fig. 5:**
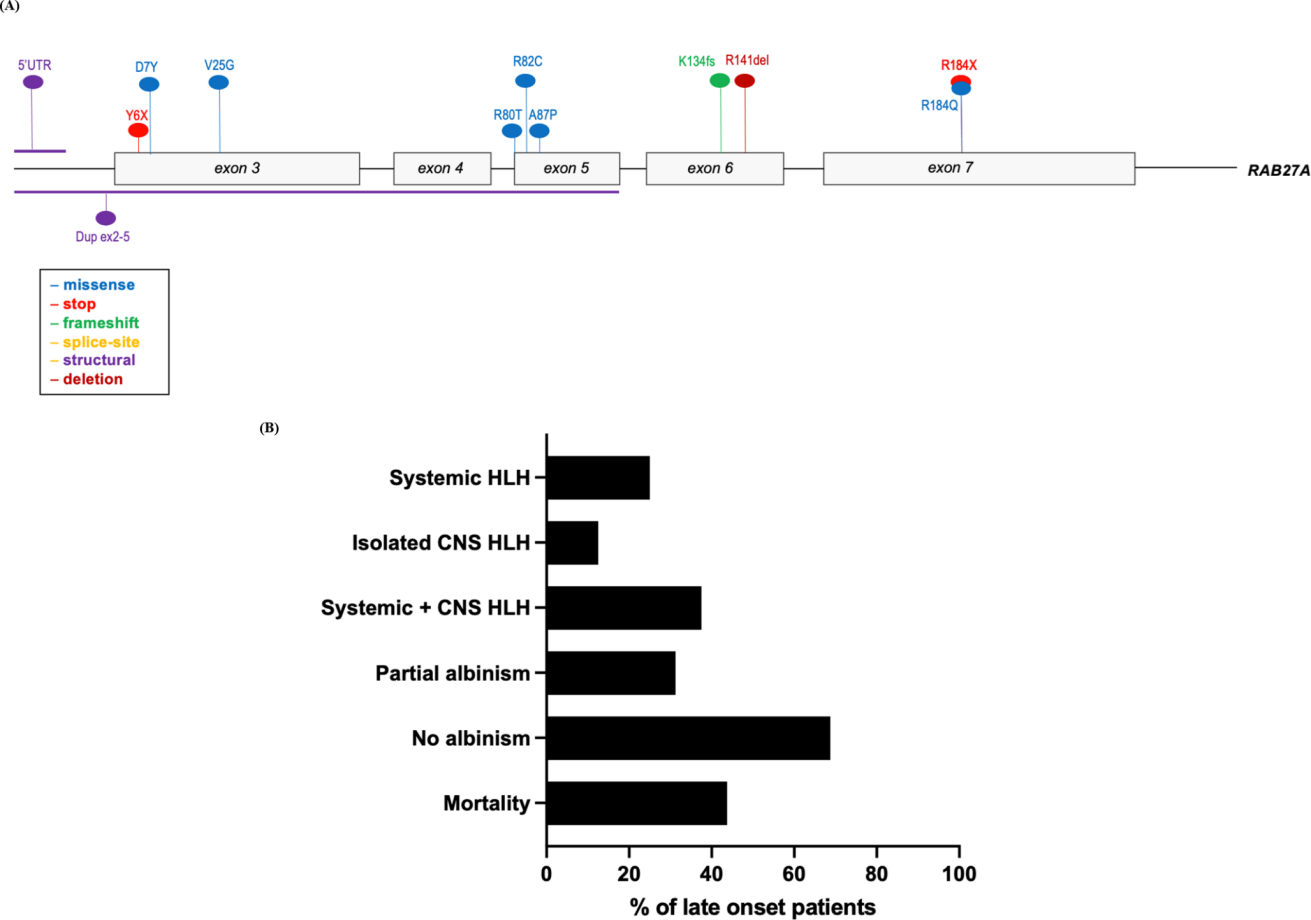
(**A**) RAB27A pathogenic variants in patients who presented at 10 years and over. The first 2 exons are non-coding (**B**) Phenotypic characteristics of patients with Griscelli syndrome type 2 presenting at age 10 or older (n = 16)

Late onset (at age ≥ 10 years) was noted in 16 cases (11%) (Fig. 5A) [13, 41]. 8 patients presented with homozygous missense hypomorphic variants (50%), while only 1 had a homozygous PTV (6%). The most common presenting feature in patients with late onset was systemic HLH (n = 10, 63%), with 8 patients also presenting with CNS HLH (50%) (Fig. 5B). Viral infections were found at HLH presentation in 8 patients (50%), with EBV implicated in 5 patients (31%). Late onset patients were more likely not to have partial albinism (n = 11, 69%) (Fig. 5B).

### Granule Release Assay

Granule release assay results were available for 46 patients. 41 of 46 patients (88%) had reduced or absent granule release. In our local cohort (P1-8), we observed normal or equivocal granule release assay, and normal or equivocal NK cytotoxicity assay for P1-5 on repeated occasions, all patients being in an extended pedigree with Qatari R82C variant. Other R82C patients were found to have reduced NK cell cytotoxicity [11, 41].

### Management Approach

For HLH-directed therapy, 22 patients received HLH-94 protocol (22/119, 18%) whilst 15 received the HLH-2004 protocol (15/119, 13%) as first-line treatments. Data specific for CNS HLH treatment were limited in patients with GS2, but individual reports describe successful regimens have included the use of high-dose corticosteroids and mycophenolate mofetil (MMF) [56, 58].

HSCT was undertaken in less than one-third of published cases (n = 44, 26%). The most common donors for HSCT were matched unrelated donors (n = 17), followed by matched related, sibling donors (n = 5). Haploidentical/mismatched related donors were used in 4 patients (15%). 5 patients were transplanted following isolated CNS HLH (5/44, 11%) with 4 patients surviving. 5 patients were transplanted pre-emptively without a prior diagnosis of HLH (n = 5, 11%) (Table 2) [39, 41, 43].

**Table 2:** Characteristics of patients who had haematopoietic stem cell transplantation (HSCT). Subsections are given as percentage of number of patients where data is available

HSCT Characteristics	Number of patients (percentage %)
**Total number of patients**	44
Age at transplant (years), median, IQR	1.8 (0.5–6.6)
Patients who received 2 HSCT	1 (2.2%)
Asymptomatic prior to HSCT	5 (11.4%)
Mortality	6 (13.6%)
**HSC source**	44
Bone marrow/stem cell	38 (86.4%)
Peripheral blood	1 (2.3%)
Cord blood	5 (11.4%)
**Donor**	26
Matched unrelated	17 (65.4%)
Matched related, sibling	5 (19.2%)
Haploidentical/ Mismatched related	4 (15.4%)
**Complications**	34
No complications	18 (52.9%)
Neurological sequelae (HLH and non-HLH related)	4 (11.8%)
Sepsis, infection	6 (17.6%)
Fatal sepsis	5 (14.7%)
Other	6 (17.6%)

### Survival and Mortality Outcomes

**Fig. 6:**
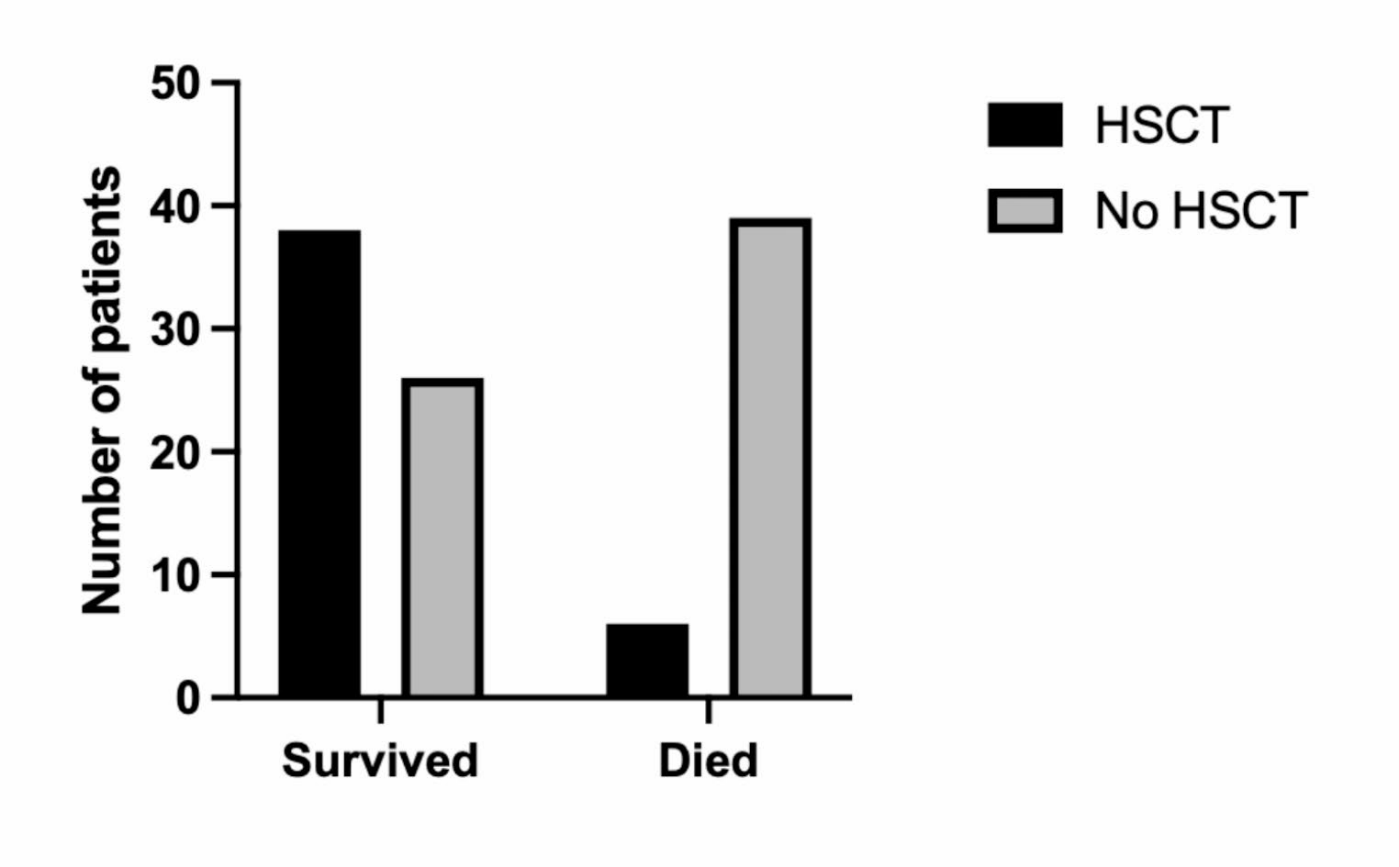
Comparison of outcomes in patients who received HSCT to those who did not

Overall, mortality was high (n = 50, 34%) in the study cohort. Data were not available to delineate specific causes of death or long-term outcomes in all patients. All patients who developed systemic HLH who did not receive HSCT shortly after, either died or were left with significant morbidity [43]. HSCT was undertaken in 44 patients with 18 having an uncomplicated post-HSCT course (41%) (Table 2). Donor information was only available in 25/44 patients (57%). Persistent neurological complications remained despite HSCT in 4 patients (9%), and fatal infections in the post-HSCT period occurred in 5 patients (11%). Among transplanted patients, 86% survived after HSCT (38/44). Of the 6 patients who died following HSCT, 5 had developed systemic and/or CNS HLH prior to HSCT (Fig. 6).

## Discussion

GS2 caused by *RAB27A* pathogenic variants, is a rare syndromic inborn error of immunity. Our study collates the largest cohort of GS2 patients to date: a total of 149 cases. The main clinical features of immunodeficiency and partial albinism usually become apparent in the first few years of life with features of fever, splenomegaly and/or HLH. Despite the distinct characteristics of GS2, immunological and/or genetic evaluation was not initiated for all patients early in life. As the differential diagnosis of partial albinism is wide and includes secondary causes such as nutritional deficiencies, a clinical or genetic diagnosis in patients is often not sought or may not be readily available [12, 72]. Diagnosis of the syndrome often occurs in the context of HLH, which can consequently lead to challenges in management and high mortality. Detection of cell surface CD107a by granule release assay is a reliable test of granule exocytosis but was only carried out in a minority of patients. Of note, we found 5 patients with *RAB27A* c.244 C > T, p.R82C variant with no detectable deficit in granule release nor NK cell killing. Given this, specific genetic testing is still recommended to confirm the diagnosis. Awareness of the condition may expedite immunological and genetic testing in children with suspected GS2 and promote monitoring for complications such as HLH.

We found biallelic PTV, missense and structural *RAB27A* pathogenic variants were distributed throughout the gene without any apparent clustering regions. We also found survival rates of patients with PTV mutations were lower compared to those with hypomorphic mutations. This suggests residual RAB27A protein expression may be protective, or that some genetic variants may cause milder disease via their specific interactions at a cellular level. Further studies are required to ascertain the molecular basis of these findings, to explore potential genotype-phenotype correlations and to gain a deeper understanding of the underlying mechanisms driving this complex and rare disorder.

In addition, we identified 3 founder mutations with distinct genotype: phenotype characteristics. Consanguinity was present in more than 65% of cases with 3 founder variants identified in predominantly European and Middle Eastern populations. Our genetic studies confirm a high prevalence of patients with a founder mutation due to co-existing consanguinity.

Heterogenous and atypical presentations can cause diagnostic delays. Consistent with previous studies, our study shows that partial albinism is not a pre-requisite feature of GS2 [6, 12]. We observed 31 patients (21%) did not have features of partial albinism and data are suggestive of favourable outcome in this group. The homozygous missense variant *RAB27A* c.244 C > T, p.R82C was most frequently encountered, but presented with atypical features such as isolated CNS HLH and normal pigmentation, the combination of which may not prompt suspicion of GS2 and lead to diagnostic delay. Patients with this founder variant are therefore subject to diagnostic delay and may present with symptoms later in life. While it may be expected to under-diagnose features of partial albinism in patients with European descent, we found that the opposite with partial albinism more likely to be present in these populations. Genetic counselling and screening should be considered for these patients with a family history, particularly in the founder regions. In addition, our findings show a larger than expected proportion of GS2 patients presenting with neurological manifestations or muscle weakness, which precede the development of either isolated CNS, or combined systemic and CNS HLH.

Sixteen patients in the cohort developed initial symptoms at age 10 years or over (11%). The most common presenting feature is systemic HLH, with only 31% having features of partial albinism. This latter feature may explain in part the late diagnosis. We also note that hypomorphic mutations tended to present later, with a slower onset of symptoms. Further functional tests are warranted to determine if residual RAB27A activity explains the late onset of symptoms.

HSCT is a potential curative therapeutic option in patients with GS2. Our study confirms the reports of *RAB27A* deficiency causing propensity to HLH and resulting in high mortality. Survival rates of patients were higher in those offered HSCT, suggesting previous assertions that pre-emptive HSCT may be a more favourable treatment option [56, 73]. The R82C variant poses a clinical dilemma regarding the timing and need of HSCT intervention in asymptomatic patients. HLH, malignancy risk, family history and a personalised approach should be considered in guiding families to consider pre-emptive HSCT. HSCT is recommended once HLH has developed for all patients due to high mortality without HSCT. HSCT-related complications were not consistently recorded, and further studies are needed to assess optimal transplant characteristics and HSCT-related outcomes. We propose the collation of follow-up data and outcomes, particularly for those undergoing HSCT in the form of a curated registry. Open access to the data will also aid the design of prospective studies, with an aim to establish optimal treatment protocols for HSCT.

As a retrospective, literature-based review, this study is limited by the phenotypic data presented by other authors, with the largest study comprising 16 patients. The study is prone to publication bias of those with unusual characteristics that may differ from a typical GS2 presentation.

In summary, this evaluation of genetic and phenotypic features of GS2 highlights a condition with high fatality secondary to HLH. The gathering of data from the literature allows us to define new significant genotype: phenotype correlations. Our data also highlight the need for an improved molecular understanding of the precise mechanisms of initiation of HLH activation, and the need for specific diagnostic testing to be available in clinical decision making. These data will help in making early diagnoses of *RAB27A* deficiency and enable timely HSCT, or in the future, specific precision therapies ultimately improving outcomes for patients with GS2.

## Electronic Supplementary Material

Below is the link to the electronic supplementary material.

Supplementary Material 1

## Data Availability

No datasets were generated or analysed during the current study.

## Abbreviations

ATG: Anti-thymocyte globulin
CTL: Cytotoxic T lymphocyte
EBV: Epstein Barr Virus
FITC: Fluorescein isothiocyanate
GRA: Granule release assay
GS2: Griscelli syndrome type 2
GTPase: Guanosine triphosphate hydrolase enzyme
HLH: Haemophagocytic lymphohistiocytosis
HSCT: Haematopoietic stem cell transplantation
IL-2: Interleukin-2
IQR: Interquartile range
MLPH: Melanophilin
MMF: Mycophenolate mofetil
MYO5A: Myosin V-A
NK: Natural killer
OMIM: Online Mendelian in Man
PHA: Phytohaemagglutinin
PTV: Protein-truncating variant
RAB27A: Ras-related protein Rab-27 A
SNP: Single nucleotide polymorphism
