# Supplementary material for: Griscelli Syndrome Type 2: Comprehensive Analysis of 149 New and Previously Described Patients with RAB27A Deficiency
Source: J Clin Immunol. 2024 Nov 28;45(1):50. doi: 10.1007/s10875-024-01842-2 (PMC11604824; doi:10.1007/s10875-024-01842-2)
Supplement: Supplementary file 1 — Supplementary Material 1 [file 10875_2024_1842_MOESM1_ESM.docx]

Supplemental Table 1: Genetic data, demographic and clinical characteristics in patients with Griscelli syndrome Type 2

| Patient (Ref) | Age of onset | Sex | Ethnicity | Consanguineous | *RAB27A* variants* (homozygous where 1 variant shown) | Type of genetic change | Systemic HLH | CNS HLH | Partial albinism | GRA | Treatment | HSCT | Known outcome/age (y) |
| --- | --- | --- | --- | --- | --- | --- | --- | --- | --- | --- | --- | --- | --- |
| P1 | 10 | M | Qatari | Yes | c.244C>T, p.R82C | Missense | Yes | Yes | No | N/E | HLH-94, Methylprednisolone, Ruxolitinib | No | Died/10 |
| P2 | 9 | F | Qatari | Yes | c.244C>T, p.R82C | Missense | No | No | No | N/E | - | No | Alive/ 11 |
| P3 | 7 | F | Qatari | Yes | c.244C>T, p.R82C | Missense | No | No | No | N/E | - | No | Alive/ 9 |
| P4 | 8 | F | Qatari | Yes | c.244C>T, p.R82C | Missense | No | No | No | N/E | - | No | Alive/ 10 |
| P5 | 4 | M | Qatari | Yes | c.244C>T, p.R82C | Missense | No | No | No | N/E | - | No | Alive/ 6 |
| P6 | 5m | M | Italian | No | c.514–518delCAAGC, p.Q172NfsX2 | PTV | No | No | Yes | Abn | - | Yes | Alive/ 10 |
| P7 | 11 | F | British | No | c.259G>C, pA87P, c550C>T p.R184* (c.h) | Missense | No | Yes | Yes | Abn | - | No | Alive/ 18 |
| P8 | 3.8 | F | Pakistani | Yes | c.239+1G>T | Splice site | Yes | Yes | Yes | Abn | HLH-94, etoposide, dexamethasone, anakinra, alemtuzumab | Yes | Alive/ 6 |
| P9 [1] | 2.5m | M | Swedish | No | c.239G>C, p.R80T, c.550C>T p.R184* (c.h) | Missense, PTV | Yes | Yes | Yes | Abn | HLH-94, ITMTX | Yes | Alive/ 7 |
| P10 [1] | 2m | F | Swedish | No | c.239G>C, p.R80T, c.550C>T p.R184* (c.h) | Missense, PTV | Yes | Yes | Yes | Abn | HLH-94 | Yes | Alive/ 2 |
| P11 [1] | 2m | F | Swedish | No | c.239G>C, p.R80T, c.550C>T p.R184* (c.h) | Missense, PTV | Yes | No | Yes | Abn | HLH-94 | Yes | Alive/ 2 |
| P12 [1] | 3m | M | Danish | No | c.550C>T, p.R184* | PTV | Yes | Yes | Yes | Abn | HLH-94, ITMTX, ITPred | Yes | Alive/ 5 |
| P13 [1] | 13 | F | Pakistani | Yes | c.550C>T, p.R184* | PTV | Yes | Yes | Yes | Abn | HLH-2004 | Yes | Alive |
| P14 [1] | 1.1 | F | German | Yes | c.148-149delinsC, p.R50QfsX35 | PTV | Yes | Yes | Yes | Abn | VP16, pred, CSA, ITMTX | Yes | Alive |
| P15 [2] | 6 | M | Iranian | Yes | c.514–518delCAAGC, p.Q172NfsX2 | PTV | Yes | Yes | nd | nd | nd | No | Died |
| P16 [2] | 3m | F | Iranian | Yes | c.514–518delCAAGC, p.Q172NfsX2 | PTV | Yes | No | Yes | nd | nd | Planned | Alive |
| P17 [2] | 8m | M | Iranian | Yes | c.514–518delCAAGC, p.Q172NfsX2 | PTV | Yes | No | nd | nd | nd | No | Died |
| P18 [2] | 5m | M | Iranian | Yes | c.514–518delCAAGC, p.Q172NfsX2 | PTV | Yes | No | nd | nd | HLH-94 | Planned | Alive |
| P19 [2] | 3 | M | Iranian | Yes | c.514–518delCAAGC, p.Q172NfsX2 | PTV | Yes | Yes | Yes | nd | nd | No | Died |
| P20 [2] | 2m | F | Iranian | Yes | c.340delA, p.I114X | PTV | Yes | No | nd | nd | Other | Planned | Alive |
| P21 [2] | 1.8 | M | Iranian | Yes | c.131T>C, p.I44T | Missense | Yes | Yes | nd | nd | Other | No | Died |
| P22 [2] | 2 | M | Iranian | Yes | c.148_149delinsC, p.R50QfsX35 | PTV | Yes | No | nd | nd | HLH-94 | nd | Alive/ 5 |
| P23 [2] | 6m | M | Iranian | Yes | c.514–518delCAAGC, p.Q172NfsX2 | PTV | Yes | Yes | nd | nd | HLH-94 | No | Died |
| P24 [3] | 3m | M | Turkish | No | c.514–518delCAAGC, p.Q172NfsX2 | PTV | Yes | No | Yes | nd | Dexamethasone, VP16, CSA | Planned | Alive/ 1.5 |
| P25 [4] | 7m | M | Turkish | Yes | nd | nd | Yes | Yes | Yes | nd | ATG, ITMTX | No | Died |
| P26 [5] | 3 | M | Brazilian | No | c.550C>T, p.R184* | PTV | No | Yes | Yes | nd | nd | No | Died |
| P27 [5] | 3.8 | M | Brazilian | No | c.550C>T, p.R184* | PTV | Yes | Yes | Yes | nd | CSA, steroids | No | Died |
| P28 [6] | 3m | F | Armenian | Yes | c.514–518delCAAGC, p.Q172NfsX2 | PTV | Yes | No | Yes | nd | ATG, ITMTX | Yes | Alive/ 2.5 |
| P29 [7] | nd | F | Arab | Yes | Deletion of exons1-5 | SV | No | Yes | Yes | nd | nd | No | Died/ 11.5 |
| P30 [7] | nd | F | Arab | Yes | Deletion of exons1-5 | SV | No | Yes | Yes | nd | nd | No | Died/ 10 |
| P31 [7] | nd | F | Arab | Yes | Deletion of exons1-5 | SV | No | Yes | Yes | nd | nd | No | Died/ 6 |
| P32 [7] | nd | M | Arab | Yes | Deletion of exons1-5 | SV | No | Yes | Yes | nd | nd | No | Died/ 2 |
| P33 [8] | 5m | M | Brazilian | No | c.352C>T, p.Q118*, c.467+1G>C (c.h) | PTV, splice site | Yes | Yes | Yes | Abn | ATG | No | Died/ 9 |
| P34 [9] | 5.8 | M | Jordanian | Yes | c.400A>G, p.K134E | Missense | Yes | Yes | Yes | nd | Other | No | Died/ 5.8 |
| P35 [10] | 8 | F | Arab | Yes | c.51delCT, p.S18WfsX15 | PTV | Yes | Yes | Yes | nd | Other | No | Died |
| P36 [11] | 3m | M | Turkish | Yes | c.514–518delCAAGC, p.Q172NfsX2 | PTV | Yes | No | Yes | nd | HLH-94 | No | Died/ 4m |
| P37 [11] | 3m | M | Turkish | Yes | c.514–518delCAAGC, p.Q172NfsX2 | PTV | Yes | No | Yes | nd | HLH-94 | No | Died/ 5m |
| P38 [12] | 5m | M | nd | Yes | c.149delG, p.R50QfsX35 | PTV | Yes | Yes | Yes | nd | Modified HLH 94 | No | Died/ 6.5 |
| P39 [12] | 9m | M | nd | Yes | c.149delG, p.R50QfsX35 | PTV | Yes | Yes | Yes | nd | Other | No | Died/ 10m |
| P40 [12] | 4m | M | nd | Yes | c.149delG, p.R50QfsX35 | PTV | Yes | Yes | Yes | nd | HLH-94 | No | Died/ 6m |
| P41 [12] | 3m | F | nd | Yes | c.149delG, p.R50QfsX35 | PTV | Yes | Yes | Yes | nd | Other | No | Died/ 6m |
| P42 [12] | 2m | F | nd | Yes | c.149delG, p.R50QfsX35 | PTV | Yes | Yes | Yes | nd | HLH-94 | Yes | Died/ 6m |
| P43 [12] | 4 | F | nd | Yes | c.217T>G, p.W73G | Missense | Yes | Yes | Yes | nd | HLH-94 | Planned | Alive/ 1 |
| P44 [12] | 4 | F | nd | Yes | c.217T>G, p.W73G | Missense | Yes | Yes | Yes | nd | HLH-94 | Yes | Alive/ 5 |
| P45 [12] | 4 | F | nd | Yes | c.346C>T, p.Q116* | PTV | Yes | Yes | Yes | nd | HLH-94 | No | Died/ 5.5 |
| P46 [13] | 4m | F | nd | Yes | c.352C>T, p.Q118*, c.467+1G>C (c.h) | PTV, splice site | Yes | No | Yes | Abn | Other | Planned | Alive |
| P47 [14] | 2.3 | F | nd | Yes | c.598C>T, p.R200* | PTV | No | Yes | Yes | nd | nd | Planned | Alive/1.5y |
| P48 [15] | 0.4m | M | nd | Yes | c.550C>T, p.R184* | PTV | No | No | Yes | nd | nd | Planned | Alive |
| P49 [16] | nd | F | Danish | No | c.550C>T, p.R184*, c.598C>T, p.R200* (c.h) | PTV | No | No | Yes | nd | nd | Yes | Alive/ 5 |
| P50 [16] | 6 | M | Afghan | Yes | c.127G>A, p.G43S | Missense | Yes | Yes | Yes | nd | nd | Yes | Alive/ 11 |
| P51 [17] | 5.5 | M | nd | Yes | c.514–518delCAAGC, p.Q172NfsX2 | PTV | Yes | No | nd | nd | nd | Yes | Alive |
| P52 [18] | nd | nd | Moroccan | nd | Deletion of exons 3-4 | SV | nd | nd | Yes | nd | nd | nd | Nd |
| P53 [19] | 1 | F | Qatari | Yes | c.244C>T, p.R82C | Missense | No | No | nd | nd | nd | No | Alive |
| P54 [19] | nd | F | Qatari | Yes | c.244C>T, p.R82C | Missense | No | No | No | nd | nd | No | Alive |
| P55 [19] | 4 | M | Qatari | Yes | c.244C>T, p.R82C | Missense | No | Yes | Yes | nd | nd | Yes | Alive |
| P56 [19] | nd | M | Qatari | Yes | c.244C>T, p.R82C | Missense | No | No | No | nd | nd | Yes | Alive |
| P57 [19] | 0.3m | M | Qatari | Yes | c.244C>T, p.R82C | Missense | No | Yes | Yes | nd | nd | No | Alive |
| P58 [19] | nd | F | Qatari | Yes | c.244C>T, p.R82C | Missense | Yes | No | No | nd | nd | No | Died/ 39 |
| P59 [19] | 10 | M | Qatari | Yes | nd | nd | Yes | Yes | No | nd | nd | No | Died/ 11 |
| P60 [19] | 9 | F | Qatari | Yes | c.244C>T, p.R82C | Missense | Yes | Yes | Yes | nd | nd | No | Died/ 12 |
| P61 [19] | 16 | M | Qatari | Yes | c.244C>T, p.R82C | Missense | No | No | No | Abn | nd | No | Alive |
| P62 [19] | 14 | M | Qatari | Yes | c.244C>T, p.R82C | Missense | No | No | No | Abn | nd | No | Alive |
| P63 [19] | 21 | M | Qatari | Yes | c.244C>T, p.R82C | Missense | Yes | No | No | nd | nd | No | Died/ 22 |
| P64 [19] | 1 | M | Qatari | Yes | c.244C>T, p.R82C | Missense | No | No | Yes | nd | nd | Yes | Alive |
| P65 [20] | 1.5m | M | Indian | No | c.550C>T, p.R184* | PTV | Yes | No | Yes | nd | nd | nd | nd |
| P66 [21] | 5.5m | F | nd | Yes | p.L26P | Missense | Yes | Yes | Yes | nd | HLH-2004 | No | Died |
| P67 [21] | 0.75m | F | nd | Yes | p.L130P | Missense | No | No | Yes | nd | nd | Yes | Died |
| P68 [21] | 7 | M | nd | Yes | c.148delA, p.R50fsX34 | PTV | Yes | No | nd | nd | HLH-2004 | No | Died |
| P69 [21] | 5 | F | nd | Yes | c.148delA, p.R50fsX34 | PTV | Yes | Yes | Yes | nd | HLH-2004 | No | Died |
| P70 [21] | 3m | M | nd | Yes | c.514–518delCAAGC, p.Q172NfsX2 | PTV | Yes | Yes | Yes | nd | HLH-2004 | Yes | Alive |
| P71 [21] | 9m | M | nd | Yes | c.514–518delCAAGC, p.Q172NfsX2 | PTV | Yes | Yes | Yes | nd | HLH-2004 | No | Alive |
| P72 [22] | 6m | M | Iranian | Yes | p.S115G | Missense | Yes | No | Yes | nd | HLH 2004 | Yes | Alive/ 1 |
| P73 [23] | 2 | M | Thai | No | c.109A>T, p.K37*, c.318T>G, p.S106R (c.h) | PTV, Missense | Yes | Yes | Yes | nd | nd | Yes | Died/ 4 |
| P74 [24] | 1.5m | nd | Turkish | Yes | c.149delG, p.R50QfsX35 | PTV | nd | nd | nd | nd | nd | nd | nd |
| P75 [24] | 1.2 | nd | Turkish | Yes | c.149delG, p.R50QfsX35 | PTV | nd | nd | nd | nd | nd | nd | nd |
| P76 [24] | 3.5m | nd | German | No | c.259G>C, p.A87P, g.20411_48243del (c.h) | Missense, SV | nd | nd | nd | nd | nd | nd | nd |
| P77 [25] | 3.8 | F | Saudi Arabia | Yes | g.55514530_55552423dup37893bp, duplication of exon2-5 | SV | Yes | Yes | Yes | Abn | nd | Planned | Alive |
| P78 [25] | 6m | M | Saudi Arabia | Yes | g.55514530_55552423dup37893bp, duplication of exon2-5 | SV | Yes | Yes | Yes | Abn | nd | nd | nd |
| P79 [25] | 5 | F | Saudi Arabia | Yes | g.55514530_55552423dup37893bp, duplication of exon2-5 | SV | Yes | Yes | Yes | Abn | nd | Yes | Alive |
| P80 [25] | 2.5m | M | Saudi Arabia | Yes | g.55514530_55552423dup37893bp, duplication of exon2-5 | SV | Yes | No | Yes | nd | nd | nd | nd |
| P81 [25] | 9m | M | Saudi Arabia | Yes | g.55514530_55552423dup37893bp, duplication of exon2-5 | SV | Yes | Yes | Yes | nd | nd | nd | nd |
| P82 [25] | 3m | F | Saudi Arabia | Yes | g.55514530_55552423dup37893bp, duplication of exon2-5 | SV | Yes | No | Yes | nd | nd | nd | Died |
| P83 [25] | 3m | M | Saudi Arabia | Yes | g.55514530_55552423dup37893bp, duplication of exon2-5 | SV | Yes | No | Yes | nd | nd | nd | Died |
| P84 [26] | 5 | F | Hispanic | nd | c.335delA, p.N112TfsX3 | PTV | Yes | Yes | Yes | nd | nd | Planned | Died |
| P85 [26] | 4m | F | Hispanic | nd | c.335delA, p.N112TfsX3 | PTV | Yes | No | Yes | nd | HLH-2004 | Yes | Alive/ 3 |
| P86 [27] | 5 | M | Korean | No | c.136T>A, p.F46I | Missense | Yes | No | Yes | nd | nd | nd | nd |
| P87 [27] | nd | M | Korean | No | c.136T>A, p.F46I | Missense | No | Yes | Yes | nd | nd | No | Died/ 7 |
| P88 [28] | 1.5 | F | Hispanic | Yes | g.53332432_53379990del, deletion exon 1 | SV | No | Yes | Yes | nd | nd | nd | nd |
| P89 [29] | 4 | M | Indian | Yes | nd | nd | Yes | Yes | Yes | nd | nd | nd | nd |
| P90 [30] | 1.6 | M | Indian | No | nd | nd | Yes | No | Yes | nd | MMF | nd | nd |
| P91 [31] | 35 | M | Turkish | Yes | c.551G>A, p.R184Q | Missense | Yes | No | Yes | Abn | Corticosteroids, IVIG, etoposide, rituximab, alemtuzumab | Planned | Died |
| P92 [32] | 3 | M | Chinese | No | c.1A>G, p.M1V | Missense | No | Yes | Yes | Abn | HLH-94 | Yes | Alive/ 3m |
| P93 [33] | 4.2 | M | nd | No | c.550C>T, p.R184* | PTV | Yes | No | Yes | nd | nd | Planned | Alive |
| P94 [34] | 1.5 | F | nd | No | c.2T>C, p.M1T, c.550C>T, p.R184* (c.h) | Missense, PTV | Yes | No | Yes | Abn | nd | Planned | Alive/ 5 |
| P95 [35] | 0.6m | M | Iranian | Yes | c.315_316delAA, p.S106FfsX18 | PTV | Yes | No | Yes | nd | HLH 94 | Planned | nd |
| P96 [36] | 3 | M | Caucasian | Yes | c.467+1G>C | Splice site | Yes | No | Yes | nd | nd | Yes | Alive/ 2 |
| P97 [37] | 1.8 | F | nd | Yes | nd | nd | No | Yes | Yes | Abn | IVIG, glucocorticoids | Yes | Alive/10 |
| P98 [38] | 14 | M | nd | nd | c.74T>G, p.V25G, c.400-401delAA, p.K134QfsX2 (c.h) | Missense, PTV | No | Yes | No | Abn | HLH-94 | Yes | Died |
| P99 [39] | 6 | F | Lithuanian | No | c.559C>T, p.R187W, 5’UTR dup-inv (c.h) | Missense, SV | Yes | Yes | No | Abn | HLH-2004 | No | Died/ 14 |
| P100 [39] | 14.5 | M | Swedish | No | 5’UTR dup-inv, c.239G>C, p.R80T (c.h) | SV, Missense | Yes | Yes | No | nd | Corticosteroids, CSA, ATG | No | Died |
| P101 [39] | 5 | M | Lithuanian | No | 5’UTR dup-inv | SV | Yes | Yes | No | Abn | HLH-2004 | Yes | Alive |
| P102 [39] | 1.75 | F | Lithuanian | No | 5’UTR dup-inv | SV | No | Yes | No | Abn | Corticosteroids, MMF | Yes | Alive/ 7 |
| P103 [39] | 10 | M | Russian | No | 5’UTR dup-inv, c.550C>T, p.R184* (c.h) | SV, PTV | No | No | No | Abn | nd | nd | Alive/ 16 |
| P104 [40] | 8 | M | Qatari | Yes | c.244C>T, p.R82C | Missense | nd | nd | No | Abn | nd | nd | Alive |
| P105 [40] | 9 | F | Qatari | Yes | c.244C>T, p.R82C | Missense | nd | Yes | No | Abn | Dexamethasone, ATG, MMF, alemtuzumab | nd | Died |
| P106 [40] | 5 | M | Qatari | Yes | c.244C>T, p.R82C | Missense | nd | nd | No | Abn | nd | nd | Alive |
| P107 [41] | 2.7 | M | nd | nd | c.428T>C, p.V143A | Missense | No | Yes | No | Abn | HLH-2004, ATG | No | Died |
| P108 [42] | 6.8m | M | Southern Europe | nd | c.422-424delGAG, p.R141_V142delinsI | Deletion | Yes | No | No | nd | nd | nd | Died |
| P109 [42] | 0.5m | M | Southern Europe | nd | c.422-424delGAG, p.R141_V142delinsI | Deletion | Yes | No | No | nd | nd | Yes | Died |
| P110 [42] | 10.7 | F | Southern Europe | nd | c.422-424delGAG, p.R141_V142delinsI | Deletion | Yes | No | No | nd | nd | Yes | Alive/ 16 |
| P111 [42] | 4 | F | Southern Europe | nd | c.422-424delGAG, p.R141_V142delinsI, c.487A>C p.S163R (c.h) | Deletion, Missense | Yes | No | No | nd | nd | Yes | Alive/ 13 |
| P112 [42] | 7.3 | M | Southern Europe | nd | c.227C>T, p. A76V, c.476A>G p. Y159C (c.h) | Missense | Yes | No | No | nd | nd | Yes | Alive/ 14 |
| P113 [42] | 5 | M | Southern Europe | nd | c.422-424delGAG, p.R141_V142delinsI, c.514–518delCAAGC, p.Q172NfsX2 (c.h) | PTV | Yes | No | No | nd | nd | Yes | Alive/ 17 |
| P114 [42] | 7m | M | Italian | nd | c.514-518delCAAGC, p.Q172NfsX2 | PTV | nd | nd | Yes | nd | nd | Yes | Alive/ 13 |
| P115 [42] | 2m | F | Italian | nd | c.550C>T, p.R184* | PTV | nd | nd | Yes | nd | nd | Yes | Alive/ 13 |
| P116 [42] | 1 | F | Italian | nd | c.149delG, p.R50QfsX35 | PTV | nd | nd | Yes | nd | nd | Yes | Alive/ 12.5 |
| P117 [42] | 11m | F | Asian | nd | c.662G>A p.C221Y | Missense | nd | nd | Yes | Abn | nd | nd | Died |
| P118 [42] | 2m | M | Italian | nd | c.514-518delCAAGC, p.Q172NfsX2 | PTV | nd | nd | Yes | nd | nd | Planned | Alive/ 2.8 |
| P119 [42] | 1m | F | Asian | nd | c.550C>T, p.R184* | PTV | nd | nd | Yes | Abn | nd | Yes | Alive/ 1.5 |
| P120 [43] | 1 | M | nd | Yes | nd | nd | Yes | No | Yes | nd | Steroids, etoposide, cyclophosphamide | Planned | Alive/ 3 |
| P121 [44] | 1.2 | F | Iranian | Yes | c.65A>G, p.K22R | Missense | Yes | No | Yes | nd | HLH-94 | Planned | Alive |
| P122 [45] | 1.8 | F | Hispanic | Yes | nd | nd | Yes | Yes | Yes | nd | nd | nd | nd |
| P123 [46] | 13 | F | Caucasian | nd | c.18_19delTG, p.Y6*, c.259G>C p.A87P (c.h) | PTV, Missense | Yes | Yes | Yes | Abn | Steroids, etoposide, IVIG, CSA, Tocilizumab | Yes | Alive/ 16 |
| P124 [47] | 5 | M | nd | No | nd | nd | Yes | No | Yes | nd | Etoposide, methylprednisolone | Yes | Alive/ 9 |
| P125 [48] | 1 | F | nd | No | g.88714G>T | Intronic | Yes | No | Yes | Abn | HLH-2004 | Yes | Alive/ 4 |
| P126 [48] | 1.4 | M | nd | nd | c.550C>T, p.R184* | PTV | Yes | No | Yes | Abn | nd | Yes | Died/ 2 |
| P127 [49] | 1 | F | nd | No | c.239+1G>T | Splice site | No | Yes | Yes | nd | Anti-epileptics, methylprednisolone | Planned | Died/ 1 |
| P128 [50] | 5 | F | Egyptian | nd | c.467+5G>A | Splice site | No | Yes | Yes | nd | nd | No | nd |
| P129 [51] | 11 | F | nd | No | nd | nd | Yes | Yes | Yes | nd | nd | Planned | Died |
| P130 [52] | 1m | F | Caucasian | No | nd | nd | Yes | No | Yes | nd | HLH-2004 | Yes | Alive/ 2 |
| P131 [53] | <6m | nd | Turkish | Yes | c.389T>C, p.L130P | Missense | Yes | No | Yes | nd | nd | nd | nd |
| P132 [53] | 8 | nd | Turkish | Yes | c.454G>C, p.A152P | Missense | Yes | No | Yes | Abn | nd | nd | nd |
| P133 [53] | 3 | M | Mauritian | No | c.550C>T, p.R184* | PTV | Yes | Yes | Yes | nd | nd | nd | nd |
| P134 [53] | 3 | M | Mauritian | No | c.550C>T, p.R184* | PTV | Yes | Yes | Yes | nd | nd | nd | nd |
| P135 [53] | 3m | nd | Turkish | Yes | c.514–518delCAAGC, p.Q172NfsX2 | PTV | Yes | No | Yes | nd | nd | nd | nd |
| P136 [53] | 3m | nd | Turkish | Yes | c.514–518delCAAGC, p.Q172NfsX2 | PTV | Yes | No | Yes | nd | nd | nd | nd |
| P137 [53] | 6m | nd | USA | Yes | c. 400_401delAA, p.K134fs | PTV | Yes | No | Yes | nd | nd | nd | nd |
| P138 [53] | <6m | nd | Turkish | Yes | c.149delG, p.R50fs | PTV | Yes | No | Yes | Abn | nd | nd | nd |
| P139 [53] | <6m | nd | Turkish | Yes | c.149delG, p.R50fs | PTV | Yes | No | Yes | nd | nd | nd | nd |
| P140 [53] | <6m | nd | Turkish | Yes | c.149delG, p.R50fs | PTV | Yes | No | Yes | nd | nd | nd | nd |
| P141 [53] | <6m | nd | Turkish | Yes | c.149delG, p.R50fs | PTV | Yes | No | Yes | nd | nd | nd | nd |
| P142 [53] | 3m | nd | North African | Yes | Deletion of exons 3-4 | SV | Yes | Yes | Yes | Abn | nd | nd | nd |
| P143 [53] | 6m | nd | North African | Yes | Deletion of exons 3-4 | SV | Yes | Yes | Yes | nd | nd | nd | nd |
| P144 [53] | 1 | nd | North African | Yes | Deletion of exons 3-4 | SV | Yes | Yes | Yes | Abn | nd | nd | nd |
| P145 [53] | 3 | nd | North African | Yes | c.239+3A>G | Splice site | Yes | No | Yes | nd | nd | nd | nd |
| P146 [53] | 6m | nd | Brazilian | Yes | c.467+1G>C | Splice site | Yes | No | Yes | nd | nd | nd | nd |
| P147 [54] | 17 | M | nd | Yes | c.19G>T, p.D7Y | Missense | No | No | No | Abn | nd | No | Alive |
| P148 [54] | 13 | M | nd | Yes | c.19G>T, p.D7Y | Missense | Yes | No | No | Abn | Steroids, etoposide, CSA | Yes | Alive/14 |
| P149 [54] | nd | F | nd | Yes | c.19G>T, p.D7Y | Missense | No | No | No | Abn | nd | No | Died /1.5 |

*RAB27A transcript GRCh37, NM_183235 or GRCh38 NM_004580.

PTV – protein-truncating variant

HLH- Haemophagocytic lymphohistiocytosis

c.h – compound heterozygous

N/E – normal or equivocal result

Abn – abnormal granule release

nd – not described

ITMTX – Intrathecal methotrexate

ITPred – Intrathecal prednisolone

Pred – prednisolone

CSA – cyclosporin A

ATG – Anti-thymocyte globulin

MMF – mycophenolate mofetil

Age is given in years, unless specified as m- months.

References:

1. Meeths, M., et al., *Clinical presentation of Griscelli syndrome type 2 and spectrum of RAB27A mutations.* Pediatr Blood Cancer, 2010. **54**(4): p. 563-72.

2. Mamishi, S., et al., *Analysis of RAB27A gene in griscelli syndrome type 2: novel mutations including a deletion hotspot.* J Clin Immunol, 2008. **28**(4): p. 384-9.

3. Onay, H., et al., *A further Turkish case of Griscelli syndrome with new RAB27A mutation.* J Am Acad Dermatol, 2008. **58**(5 Suppl 1): p. S115-6.

4. Aslan, D., et al., *Griscelli syndrome: description of a case with Rab27A mutation.* Pediatr Hematol Oncol, 2006. **23**(3): p. 255-61.

5. Meschede, I.P., et al., *Griscelli syndrome-type 2 in twin siblings: case report and update on RAB27A human mutations and gene structure.* Braz J Med Biol Res, 2008. **41**(10): p. 839-48.

6. Schuster, F., et al., *Griscelli syndrome: report of the first peripheral blood stem cell transplant and the role of mutations in the RAB27A gene as an indication for BMT.* Bone Marrow Transplant, 2001. **28**(4): p. 409-12.

7. Anikster, Y., et al., *Evidence that Griscelli syndrome with neurological involvement is caused by mutations in RAB27A, not MYO5A.* Am J Hum Genet, 2002. **71**(2): p. 407-14.

8. Bizario, J.C., et al., *Griscelli syndrome: characterization of a new mutation and rescue of T-cytotoxic activity by retroviral transfer of RAB27A gene.* J Clin Immunol, 2004. **24**(4): p. 397-410.

9. Masri, A., et al., *Griscelli syndrome type 2: a rare and lethal disorder.* J Child Neurol, 2008. **23**(8): p. 964-7.

10. Aksu, G., et al., *Griscelli syndrome without hemophagocytosis in an eleven-year-old girl: expanding the phenotypic spectrum of Rab27A mutations in humans.* Am J Med Genet A, 2003. **116a**(4): p. 329-33.

11. Sarper, N., et al., *A rare syndrome in the differential diagnosis of hepatosplenomegaly and pancytopenia: report of identical twins with Griscelli disease.* Ann Trop Paediatr, 2003. **23**(1): p. 69-73.

12. Sanal, O., et al., *Griscelli disease: genotype-phenotype correlation in an array of clinical heterogeneity.* J Clin Immunol, 2002. **22**(4): p. 237-43.

13. Gazit, R., et al., *NK cytotoxicity mediated by CD16 but not by NKp30 is functional in Griscelli syndrome.* Blood, 2007. **109**(10): p. 4306-12.

14. Rajadhyax, M., et al., *Neurological presentation of Griscelli syndrome: obstructive hydrocephalus without haematological abnormalities or organomegaly.* Brain Dev, 2007. **29**(4): p. 247-50.

15. Sheela, S.R., M. Latha, and S.J. Injody, *Griscelli syndrome: Rab 27a mutation.* Indian Pediatr, 2004. **41**(9): p. 944-7.

16. Westbroek, W., et al., *A novel missense mutation (G43S) in the switch I region of Rab27A causing Griscelli syndrome.* Mol Genet Metab, 2008. **94**(2): p. 248-54.

17. Aricò, M., et al., *Successful treatment of Griscelli syndrome with unrelated donor allogeneic hematopoietic stem cell transplantation.* Bone Marrow Transplant, 2002. **29**(12): p. 995-8.

18. Westbroek, W., et al., *Rab27b is up-regulated in human Griscelli syndrome type II melanocytes and linked to the actin cytoskeleton via exon F-Myosin Va transcripts.* Pigment Cell Res, 2004. **17**(5): p. 498-505.

19. Al-Sulaiman, R., et al., *A founder RAB27A variant causes Griscelli syndrome type 2 with phenotypic heterogeneity in Qatari families.* Am J Med Genet A, 2020. **182**(11): p. 2570-2580.

20. Ariffin, H., et al., *Griscelli syndrome.* Med J Malaysia, 2014. **69**(4): p. 193-4.

21. Durmaz, A., et al., *Molecular analysis and clinical findings of Griscelli syndrome patients.* J Pediatr Hematol Oncol, 2012. **34**(7): p. 541-4.

22. Shamsian, B.S., et al., *A novel RAB27A mutation in a patient with Griscelli syndrome type 2.* J Investig Allergol Clin Immunol, 2010. **20**(7): p. 612-5.

23. Yeetong, P., K. Suphapeetiporn, and V. Shotelersuk, *Mutation analysis and prenatal diagnosis of a family with Griscelli syndrome type 2: two novel mutations in the RAB27A gene.* World J Pediatr, 2017. **13**(4): p. 392-394.

24. Zur Stadt, U., et al., *Mutation spectrum in children with primary hemophagocytic lymphohistiocytosis: molecular and functional analyses of PRF1, UNC13D, STX11, and RAB27A.* Hum Mutat, 2006. **27**(1): p. 62-8.

25. Grandin, V., et al., *A RAB27A duplication in several cases of Griscelli syndrome type 2: An explanation for cases lacking a genetic diagnosis.* Hum Mutat, 2017. **38**(10): p. 1355-1359.

26. Russ, A., et al., *Griscelli Type 2 Syndrome and Hemophagocytic Lymphohistiocytosis: Sisters With the Same Mutation but Different Presentations.* J Pediatr Hematol Oncol, 2019. **41**(6): p. 473-477.

27. Mishra, K., et al., *Griscelli syndrome type 2: a novel mutation in RAB27A gene with different clinical features in 2 siblings: a diagnostic conundrum.* Korean J Pediatr, 2014. **57**(2): p. 91-5.

28. Vincent, L.M., et al., *Novel 47.5-kb deletion in RAB27A results in severe Griscelli Syndrome Type 2.* Mol Genet Metab, 2010. **101**(1): p. 62-5.

29. Rajyalakshmi, R. and R.N. Chakrapani, *Griscelli syndrome type 2: A rare and fatal syndrome in a South Indian boy.* Indian J Pathol Microbiol, 2016. **59**(1): p. 113-6.

30. Minocha, P., et al., *Griscelli syndrome subtype 2 with hemophagocytic lympho-histiocytosis: A case report and review of literature.* Intractable Rare Dis Res, 2017. **6**(1): p. 76-79.

31. Zondag, T.C.E., et al., *Novel RAB27A Variant Associated with Late-Onset Hemophagocytic Lymphohistiocytosis Alters Effector Protein Binding.* J Clin Immunol, 2022. **42**(8): p. 1685-1695.

32. Zhang, Q., et al., *Successful rescue of a lethal Griscelli syndrome type 2 presenting with neurological involvement and hemophagocytic lymphohistiocytosis: a case report.* BMC Pediatr, 2021. **21**(1): p. 253.

33. Tewari, N., et al., *Oral features of Griscelli syndrome type II: A rare case report.* Spec Care Dentist, 2018. **38**(6): p. 421-425.

34. Gotesman, R., et al., *Cutaneous granulomas as the presenting manifestation of Griscelli syndrome type 2.* Pediatr Dermatol, 2021. **38**(1): p. 194-197.

35. Nodehi, H., et al., *Neonatal Onset of Hemophagocytic Lymphohistiocytosis Due to Prenatal Varicella-Zoster Infection in a Neonate with Griscelli Syndrome Type 2.* Iran J Allergy Asthma Immunol, 2022. **21**(4): p. 488-493.

36. Gironi, L.C., et al., *Congenital Hypopigmentary Disorders with Multiorgan Impairment: A Case Report and an Overview on Gray Hair Syndromes.* Medicina (Kaunas), 2019. **55**(3).

37. Castaño-Jaramillo, L.M., et al., *Diagnostic and therapeutic caveats in Griscelli syndrome.* Scand J Immunol, 2021. **93**(6): p. e13034.

38. Woodward, K.E., et al., *Considering immunologic and genetic evaluation for HLH in neuroinflammation: A case of Griscelli syndrome type 2 with neurological symptoms and a lack of albinism.* Pediatr Blood Cancer, 2020. **67**(8): p. e28312.

39. Tesi, B., et al., *A RAB27A 5' untranslated region structural variant associated with late-onset hemophagocytic lymphohistiocytosis and normal pigmentation.* J Allergy Clin Immunol, 2018. **142**(1): p. 317-321.e8.

40. Netter, P., et al., *A novel Rab27a mutation binds melanophilin, but not Munc13-4, causing immunodeficiency without albinism.* J Allergy Clin Immunol, 2016. **138**(2): p. 599-601.e3.

41. Ohishi, Y., et al., *Griscelli Syndrome Type 2 Sine Albinism: Unraveling Differential RAB27A Effector Engagement.* Front Immunol, 2020. **11**: p. 612977.

42. Cetica, V., et al., *Patients with Griscelli syndrome and normal pigmentation identify RAB27A mutations that selectively disrupt MUNC13-4 binding.* J Allergy Clin Immunol, 2015. **135**(5): p. 1310-8.e1.

43. Sefsafi, Z., et al., *Macrophage activation syndrome associated with griscelli syndrome type 2: case report and review of literature.* Pan Afr Med J, 2018. **29**: p. 75.

44. Ohbayashi, N., et al., *Functional characterization of two RAB27A missense mutations found in Griscelli syndrome type 2.* Pigment Cell Melanoma Res, 2010. **23**(3): p. 365-74.

45. Emanuel, P.O., L.J. Sternberg, and R.G. Phelps, *Griscelli syndrome.* Skinmed, 2007. **6**(3): p. 147-9.

46. Messinger, Y.H., et al., *Delayed diagnosis of Griscelli syndrome type 2 with compound heterozygote RAB27A variants presenting with pulmonary failure.* Pediatr Hematol Oncol, 2021. **38**(6): p. 593-601.

47. Rossi, A., et al., *Griscelli syndrome type 2: long-term follow-up after unrelated donor bone marrow transplantation.* Dermatology, 2009. **218**(4): p. 376-9.

48. Gupta, D., et al., *Hematopoietic stem cell transplantation in children with Griscelli Syndrome type 2: Experience and outcomes.* Indian J Pathol Microbiol, 2019. **62**(2): p. 279-282.

49. Panigrahi, I., et al., *Seizure as the presenting manifestation in Griscelli syndrome type 2.* Pediatr Neurol, 2015. **52**(5): p. 535-8.

50. Lee, J.Y.W., et al., *Further evidence for genotype-phenotype disparity in Griscelli syndrome.* Br J Dermatol, 2017. **176**(4): p. 1086-1089.

51. Szczawinska-Poplonyk, A., et al., *Pulmonary lymphomatoid granulomatosis in Griscelli syndrome type 2.* Viral Immunol, 2011. **24**(6): p. 471-3.

52. Hinson, A., et al., *Congenital hemophagocytic lymphohistiocytosis presenting as thrombocytopenia in a newborn.* J Pediatr Hematol Oncol, 2015. **37**(4): p. 300-3.

53. Ménasché, G., et al., *Mutations in RAB27A cause Griscelli syndrome associated with haemophagocytic syndrome.* Nat Genet, 2000. **25**(2): p. 173-6.

54. Brauer, N., et al., *Immunodeficiency with susceptibility to lymphoma with complex genotype affecting energy metabolism (FBP1, ACAD9) and vesicle trafficking (RAB27A).* Front Immunol, 2023. **14**: p. 1151166.
